# Antimicrobial Properties of Mesenchymal Stem Cells: Therapeutic Potential for Cystic Fibrosis Infection, and Treatment

**DOI:** 10.1155/2016/5303048

**Published:** 2016-01-26

**Authors:** Morgan T. Sutton, David Fletcher, Santosh K. Ghosh, Aaron Weinberg, Rolf van Heeckeren, Sukhmani Kaur, Zhina Sadeghi, Adonis Hijaz, Jane Reese, Hillard M. Lazarus, Donald P. Lennon, Arnold I. Caplan, Tracey L. Bonfield

**Affiliations:** ^1^Department of Pediatrics, Case Western Reserve University, Cleveland, OH 44106, USA; ^2^National Center of Regenerative Medicine, Case Western Reserve University, Cleveland, OH 44106, USA; ^3^School of Dentistry, Case Western Reserve University, Cleveland, OH 44106, USA; ^4^Department of Urology, Case Western Reserve University, Cleveland, OH 44106, USA; ^5^Department of Cancer Biology, Case Western Reserve University, Cleveland, OH 44106, USA; ^6^CTSC Cell Therapy Laboratory, Case Western Reserve University, Cleveland, OH 44106, USA; ^7^Department of Biology, Case Western Reserve University, Cleveland, OH 44106, USA; ^8^Skeletal Research Center, Case Western Reserve University, Cleveland, OH 44106, USA

## Abstract

Cystic fibrosis (CF) is a genetic disease in which the battle between pulmonary infection and inflammation becomes the major cause of morbidity and mortality. We have previously shown that human MSCs (hMSCs) decrease inflammation and infection in the* in vivo* murine model of CF. The studies in this paper focus on the specificity of the hMSC antimicrobial effectiveness using* Pseudomonas aeruginosa* (gram negative bacteria) and* Staphylococcus aureus* (gram positive bacteria). Our studies show that hMSCs secrete bioactive molecules which are antimicrobial* in vitro* against* Pseudomonas aeruginosa, Staphylococcus aureus,* and* Streptococcus pneumonia*, impacting the rate of bacterial growth and transition into colony forming units regardless of the pathogen. Further, we show that the hMSCs have the capacity to enhance antibiotic sensitivity, improving the capacity to kill bacteria. We present data which suggests that the antimicrobial effectiveness is associated with the capacity to slow bacterial growth and the ability of the hMSCs to secrete the antimicrobial peptide LL-37. Lastly, our studies demonstrate that the tissue origin of the hMSCs (bone marrow or adipose tissue derived), the presence of functional cystic fibrosis transmembrane conductance regulator (CFTR: human,* Cftr*: mouse) activity, and response to effector cytokines can impact both hMSC phenotype and antimicrobial potency and efficacy. These studies demonstrate, the unique capacity of the hMSCs to manage different pathogens and the significance of their phenotype in both the antimicrobial and antibiotic enhancing activities.

## 1. Introduction

Cystic fibrosis (CF) is an inherited, fatal disease in which the cystic fibrosis transmembrane conductance regulator (CFTR: human) gene is mutated, resulting in defective CFTR receptor sodium chloride pump activity. Inefficient CFTR function results in the dysregulated balance of sodium and chloride ions across epithelial cell membranes, increasing mucous viscosity which ultimately contributes to a variety of anomalies including pulmonary infection and gastrointestinal obstruction [1, 2]. Despite new developments in small molecule correctors and CFTR expression enhancers, pulmonary bronchiectasis, bacterial colonization, and the ensuing inflammatory response continue to contribute to lung congestion, pulmonary failure, and death in CF patients [2, 3]. Human mesenchymal stem cells (hMSCs) secrete bioactive molecules that are anti-inflammatory, antimicrobial, angiogenic, chemotactic, antiapoptotic, and antiscarring [4, 5] and have been investigated in several lung disease models including asthma, interstitial pulmonary fibrosis, and adult respiratory distress syndrome [4, 6, 7]. We have previously published the potential therapeutic efficacy of hMSCs in the preclinical murine model of CF lung infection and inflammation [8]. In these studies, wild type (WT) and CFTR deficient mice (CF) were chronically infected with* Pseudomonas aeruginosa* and followed for several days for clinical score, survival, and weight loss kinetics post-MSC treatment. In this* in vivo* model of CF lung infection and inflammation, hMSCs decreased the bacterial burden and thereby enhanced the ability of the CF lung to resolve the infection potentially through changes in the* in vivo* production of the antimicrobial peptide LL-37. These results were similar to the studies done with hMSCs in sepsis [9].

Although* Pseudomonas aeruginosa* is the most prevalent bacteria in CF infections and the one most studied in the context of CF, it is not the only bacteria colonizing CF patients. Other organisms such as* Streptococcus pneumoniae *and* Staphylococcus aureus *also create a complex pulmonary niche and microbiome for the CF lung [10, 11]. Our studies were designed to begin to understand the antimicrobial activity of the hMSCs and the mechanisms associated with the antimicrobial specificity of the hMSCs and their products. We show that the antimicrobial potency of the hMSCs can impact the outcome of infections associated with not only* Pseudomonas aeruginosa *but also* Staphylococcus aureus* and* Streptococcus pneumoniae. *Further, the hMSCs enhance antibiotic potency against each of these pathogens by slowing pathogen growth rate and the production of the antimicrobial peptide LL-37. Our studies explore the impact of CFTR activity on hMSC action, the tissue source of hMSCs, and whether the hMSCs can be optimized for antimicrobial potency and efficacy. In summary, our studies show that hMSC antimicrobial potential depends on their phenotype, which can be optimized for complex multicomponent therapeutic applications.

## 2. Methods

### 2.1. Cell Preparations

This study was approved by the Case Western Reserve University and University Hospitals of Cleveland Institutional Review Board (IRB) and Ethics Committees. Human posterior iliac crest bone marrow samples were obtained after written informed consent from paid volunteers under IRB, #CASE12Z05. Human MSCs (hMSCs) were expanded in* ex vivo* culture per previously published methods [12, 13]. Human adipose derived MSCs (hADSC) were isolated from elective abdominal liposuction discarded tissue of obese (25 < BMI < 30), otherwise healthy, volunteers also under an institutionally approved IRB (#UH 03-11-22). The dispersed adipose tissue was rinsed with phosphate buffered saline (PBS) and was then incubated in a solution containing 0.1% collagenase type IA (Aldrich-Sigma, St. Louis, MO) for 1 hour at 37°C while being shaken vigorously. The digested tissue was centrifuged at 6000 g for 10 min at room temperature. The pellet at the bottom of tube (stromal vascular fraction, SVF) was then suspended in Dulbecco's-Modified Eagle's Medium (DMEM) supplemented with 10% fetal bovine serum (FBS, nonselected, Biomed Corporation, Foster City, CA) and 10 mL of F-12 nutritional media (DMEM : F-12, 1 : 1) and plated at a density of 1000 cells/mm^2^. The culture dish was placed in a 5% CO_2_ incubator for 6–8 days to allow for the formation of MSC colonies, which were then trypsinized and propagated. MSCs were validated for the ability to produce chondrocytes and by flow cytometry as previously described [14].* hMSC supernatants*: hMSCs were grown to confluence and put into serum-free, antibiotic-free conditions for 3 days prior to harvesting the conditioned medium from the confluent cells. Controls of medium alone were used in all of the experiments. All hMSC preparations were utilized at either cell passage 2 or passage 3 and were validated for the ability to produce chondrocytes and phenotyped by flow cytometry as described previously [15]. We did not test the adipose and osteodifferentiation of the hMSCs since our studies specifically evaluated the predifferentiation potential of the hMSCs to be bioactive antimicrobial contributors prior to differentiation. The chondrocyte protocols are used to define the healthy and potential functionality of the preparation.

### 2.2. Bacteria

*Pseudomonas aeruginosa* (PA M5715) is a clinical strain obtained with consent [16].* Staphylococcus aureus* (ATCC #25923) and* Streptococcus pneumoniae* (ATCC #49619) were grown in Lysogeny Broth (LB), using each of the pathogens in log phase of growth defined by their growth curves [15, 17, 18]. Bacteria were also evaluated for viability and growth profiles prior to utilization in the studies. Bacteria were grown in a BSCL-2 facility which was approved by the Case Western Reserve University Department of Laboratory Safety.

### 2.3. Animal Studies

All animal studies were done with institutional approval (Case Western Reserve University IACUC: 2014-0093) with each study being done at least 4 times. Mice were used between the ages of 8 weeks to 12 weeks without distinction between sexes. All mice were housed in our CF Lung Infection and Inflammatory Core procedure room prior to and during the entire course of the experiments. Animals were housed in groups of 5 mice/cage, with husbandry maintained by both the Core and the Case Western Reserve Animal Resource Center. Cystic fibrosis transmembrane conductance regulator deficient animals (*Cftr* ^*tm1Kth*^,* Cftr*, *n* = 10/study) and congenic background controls (C57BL/6J, WT, *n* = 10/study) were anesthetized with IACUC approved ketamine analgesic to minimize discomfort and to maintain pulmonary physiology. Agarose beads impregnated with the pathogens were inoculated into the lungs of the mice using transtracheal administration. All animals received 10^5^ *Pseudomonas aeruginosa, *or* Staphylococcus aureus* as defined by postculture of the agarose beads. 24 hours after infection, mice were infused with 10^6^ adult bone marrow derived hMSCs through the retroorbital sinus. We have found that this route allows for direct deposition into the lung [8]. Animals were followed with daily weights and clinical scores out to 10 days, with an assessment within 2 hours of the time of the initial infection. Mice were housed in our separate animal procedure room monitored by the Core Staff and the Animal Resource Center according to the Case Western Reserve University guidelines. The lung bacteria load was evaluated by bronchoalveolar lavage (BAL) for BAL fluid and whole tissue lung homogenates for* Pseudomonas aeruginosa* or* Staphylococcus aureus* colony forming units (CFUs) streaking both tissue sources onto Tryptic Soy Agarose (TSA) plates (20 mL agar/plate) over a variety of dilutions cultured for 24 hours. Each of the models were used to investigate how the hMSCs affected the response of the murine CF model to gram negative (*Pseudomonas aeruginosa*) and gram positive (*Staphylococcus aureus*) organisms, respectively. Animals were identified by numbers to minimize bias between experimental groups. We did not do* in vivo* studies with* Streptococcus pneumonia*, although a different pathogen is also a gram positive organism and would be a redundant use of animals.

### 2.4. Bacteriology

Bacteria were combined with 2 mL of hMSC supernatants derived from several unidentified donors (*n* ≥ 4) with or without the presence of the antibiotics geneticin, tobramycin, or ceftazadine (100 *μ*g/mL antibiotic concentrations), using individual cultures for each donor. After incubation, the bacteria were serially diluted in phosphate buffered saline (PBS) to dilutions of 10^−6^–10^−9^ followed by growth on Tryptic Soy Agar plates (10 *μ*L/column, for* Pseudomonas aeruginosa* and* Staphylococcus aureus*). MacConkey plates were utilized for* Streptococcus pneumoniae*. Bacteria CFUs were counted after 24 hours for the time-point specimens. For all of the studies, 1 mL aliquots of the different bacterial combinations were evaluated for the number of live and growing bacteria measured by ATP luminescence with Bac-Titer Glo assays (Madison, WI) using an ATP (pg/mL) standard curve and quantified using 5-parameter statistics. 1 *μ*M ATP was prepared in culture medium. 10-fold serial dilutions in Tryptic Soy Broth (TSB) were used for standard curve. 100 *μ*L of sample was added to luminometer plate with 100 *μ*L of Glo substrate with analysis using Nikon Luminometer (luciferase, 10-second analysis, no injection).

### 2.5. Secreted Antimicrobial Peptides

Supernatants from cultured hMSCs were evaluated for LL-37, human beta defensins (hBD) hBD-2, and hBD-3 using ELISA methodology. The levels of LL-37 were determined utilizing a commercially available kit (Hycult, Biotech, Plymouth Meeting PA), following manufacturing instructions. The ELISA assays for human beta defensin (hBD) type 2 and 3 (hBD-2, hBD-3, resp.) were done following previously published methods [19, 20]. Data is expressed as mean (pg/mL ± SEM, *n* ≥ 4).

### 2.6. Data Evaluation and Statistics

Data is expressed as mean ± standard error of the mean (SEM) through nonparametric Mann-Whitney tests. Analysis of variances and linear correlations were performed using GraphPad Prism 6 (La Jolla, CA). Percent error was calculated through use of Student's *t*-tests or analysis of variance as indicated. All significant values were defined as the value of *P* ≤ 0.05. In some cases *P* values which are close to the 5% confidence interval are given to demonstrate trends towards significance. Analyses of log or square-root transformation were used to compare experimental conditions at a single point with paired *t*-tests and slopes over time, using different stem cell donors as replicates. In the animal infection models, the bacteria counts were compared using stratified log-rank tests, with the stem cell donors being correlated with the outcomes of the* in vivo* resolution of bacteria load posttreatment. When comparing antimicrobial LL-37 production or* Cftr* gene expression levels, we used paired *t*-tests, assuming standard deviations of 0.75 on the log_2_ scale as suggested by Simon [21, 22]. A 2.8-fold difference in expression can be detected with 80% power with a two-sided* t*-test at the 0.01 significance level. When the data could not be transformed to normality, a nonparametric van-Elteren test (van Elteren, 1960) [23, 24] was used to compare groups, stratifying on the donors. All statistical analyses were done with the assistance of the Statistical Resource Center, Department of Pediatrics.

## 3. Results

### 3.1. *In Vivo* Antimicrobial Potency and Efficacy

**Figure 1:**
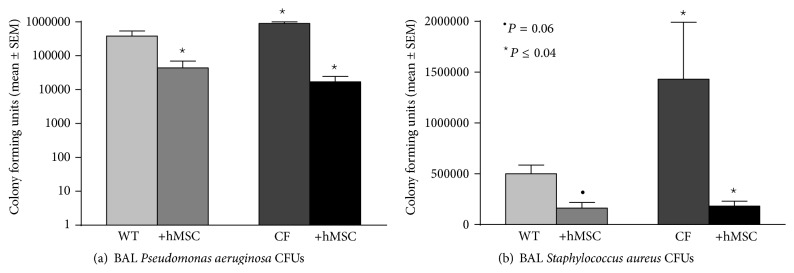
hMSCs in the PA (a) and SA (b) infection model:* Cftr* ^*tm1Kth*^ (CF) and wild type (WT) controls were infected with 10^5^ CFUs of either* Pseudomonas aeruginosa* or* Staphylococcus aureus* impregnated into agarose beads to generate chronic gram negative or gram positive chronic infection models in CF. hMSCs were administered on day 1, 24 hours after infection. Mice were followed up to 10 days and were then euthanized for bacteria burden (BAL CFUs+ whole lung homogenate CFUs, *n* = 4 experiments with 10 animals in each group). hMSCs decreased bacteria burden (*P* ≤ 0.05) in response to both pathogens.

C57BL/6J and* Cftr* ^*tm1Kth*^ knockout mice were infected with either* Pseudomonas aeruginosa* or* Staphylococcus aureus* followed 24 hours later with an infusion of 10^6^ bone marrow derived hMSCs through the retroorbital sinus. The mice were then monitored for 10 days prior to determining their infection status. Any animals that died during the study were documented and held for bacteriology by necropsy. BAL from the* Cftr* ^*tm1Kth*^ mice (CF) had significantly higher CFUs (in log scale) than the C57BL/6J (WT) regardless of whether infections were associated with* Pseudomonas aeruginosa* (Figure 1(a); *P* ≤ 0.05 for each, *n* = 10) or* Staphylococcus aureus* (Figure 1(b); *P* ≤ 0.05 for each, *n* = 10). In either of the two infection models, the CFUs were significantly decreased with hMSC therapy (*P* ≤ 0.05 for each, *n* = 4 different hMSC preparations).

### 3.2. MSCs Potency and Efficacy on Bacterial Growth

**Figure 2:**
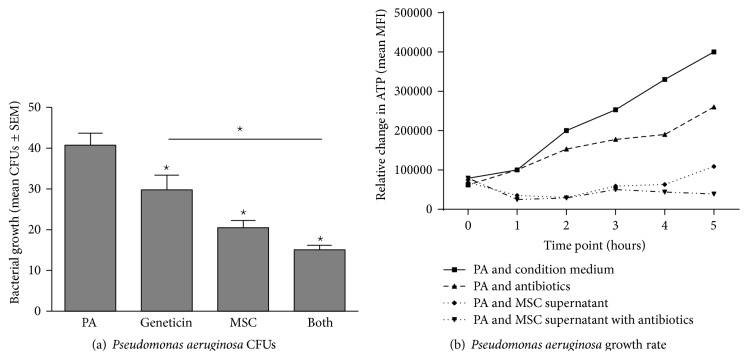
MSCs products decrease* Pseudomonas aeruginosa* growth. Bone marrow derived hMSCs supernatants were cultured with different dosages of* Pseudomonas aeruginosa* with and without the addition of geneticin (100 *μ*g/mL). Aliquots of the bacteria were streaked onto TSA plates for CFUs (a) or evaluated for ATP production (b). hMSC supernatants (*n* = 8 different donors) significantly decreased both* Pseudomonas aeruginosa* growth kinetics (*P* ≤ 0.05) and CFUs (*P* ≤ 0.05). Geneticin was used as a positive control which also significantly decreased both CFUs (*P* ≤ 0.05) and growth rate (*P* ≤ 0.05) which was enhanced by the addition of hMSCs (*P* ≤ 0.05 versus antibiotic alone for both CFUs and growth kinetics). PA =* Pseudomonas aeruginosa* growth without treatment. Geneticin = treatment with the antibiotic geneticin, +hMSCs = treatment with hMSC derived supernatant, and Both = treatment with both hMSC supernatants and geneticin.

**Figure 3:**
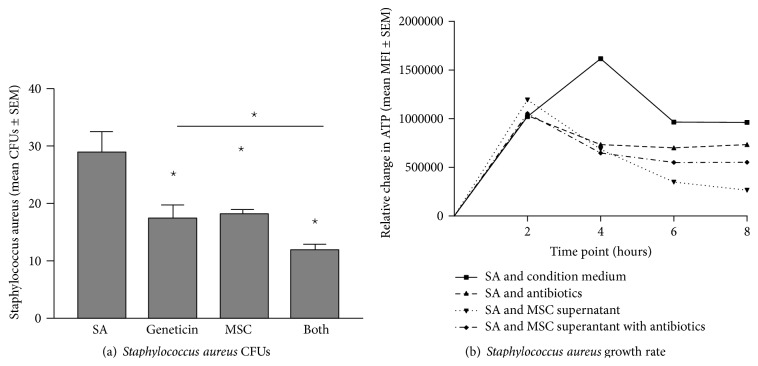
MSCs and their products alter* Staphylococcus aureus* growth. Bone marrow derived hMSCs supernatants were cultured with different dosages of* Staphylococcus aureus* with and without the addition of geneticin (100 *μ*g/mL). Aliquots of the bacteria were streaked onto TSA plates for CFUs (a) or evaluated for ATP production (b). hMSC supernatants (*n* = 8 different donors) significantly decreased both* Staphylococcus aureus* CFUs (*P* ≤ 0.05) and growth kinetics (*P* ≤ 0.05). Geneticin was used as a positive control which also significantly decreases both CFUs (*P* ≤ 0.05) and growth rate (*P* ≤ 0.05) which was enhanced by the addition of hMSCs (*P* ≤ 0.05 versus antibiotic alone for both CFUs and growth rate). SA =* Staphylococcus aureus* growth without treatment. Geneticin = treatment with the antibiotic geneticin, +hMSCs = treatment with hMSC derived supernatant, and Both = treatment with both hMSC supernatants and geneticin.

**Figure 4:**
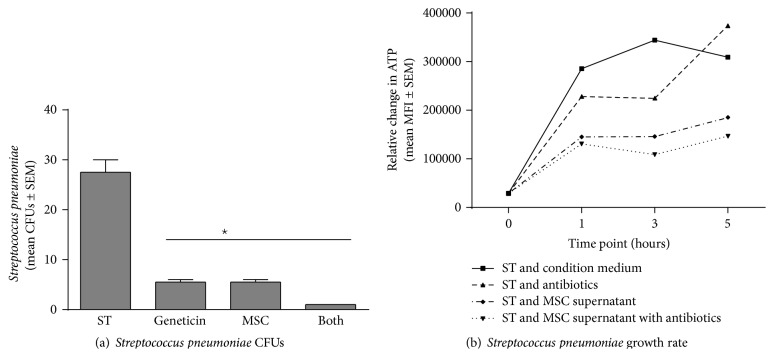
hMSCs and their products on* Streptococcus pneumoniae* growth. Bone marrow derived hMSCs supernatants were cultured with different dosages of* Streptococcus pneumoniae* with and without the addition of geneticin (100 *μ*g/mL). Aliquots of the bacteria were streaked onto MacConkey plates for CFUs (a) or evaluated for ATP production (b). hMSC supernatants (*n* = 8 different donors) significantly decreased both* Streptococcus pneumoniae* CFUs ((a), *P* ≤ 0.05) and growth rates ((b), *P* ≤ 0.05). Geneticin was used as a positive control, decreasing both CFUs (*P* ≤ 0.05) and growth rates (*P* ≤ 0.05). hMSC supernatants decreased CFUs and enhanced antibiotic sensitivity when measuring CFUs. However, hMSCs supernatants had minimal antibiotic enhancing effect on* Streptococcus pneumonia* growth rate (b). ST =* Streptococcus pneumoniae* growth without treatment. Geneticin = treatment with the antibiotic geneticin, +hMSCs = treatment with hMSC derived supernatant, and Both = treatment with both MSC supernatants and geneticin.

Bone marrow derived hMSC supernatants were evaluated for their ability to alter* Pseudomonas aeruginosa* (Figure 2),* Staphylococcus aureus* (Figure 3), and* Streptococcus pneumoniae* (Figure 4) growth rate and survival* in vitro* using geneticin antibiotics (100 *μ*g/mL) as a positive control.

#### 3.2.1. *Pseudomonas aeruginosa* (Figure 2)

In evaluating the impact of bone marrow derived hMSC secreted soluble factors on* Pseudomonas aeruginosa* growth (Figure 2(a)), we found that hMSC supernatants decreased* Pseudomonas aeruginosa* numbers from 41 ± 3 CFUs (mean ± SEM, *n* = 4 different hMSC donor preparations) to 21 ± 2 CFUs (*P* < 0.05). Geneticin decreased* Pseudomonas aeruginosa* growth to 30 ± 4 CFUs, which, when combined with hMSC supernatants, decreased* Pseudomonas aeruginosa* growth to 15 ± 1 (*P* < 0.05). To determine the impact of the hMSC supernatants on the growth rate of* Pseudomonas aeruginosa*, we measured bacterial ATP production (mean MFI ± SEM, *n* = 4) versus time (up to 24 hours) focusing on the slope and duration of the hMSC effectiveness.* Pseudomonas aeruginosa* without hMSC supernatants had a slope of 36 ± 6 (Figure 2(b)). Treatment of the* Pseudomonas aeruginosa* with the geneticin slowed the growth rate of the bacteria to a slope of 26 ± 6, while hMSC supernatant treatment of the pathogen decreased the slope to −51 ± 10 which was significantly less when compared to the bacterial growth rate without treatment (*P* < 0.05, *n* = 4). When combining the geneticin and MSC supernatants there was an additive effect, decreasing the* Pseudomonas aeruginosa* growth rate to a slope of −68 ± 11, statistically less than either hMSC or geneticin alone (*P* < 0.05 for each, *n* = 4).

#### 3.2.2. *Staphylococcus aureus* (Figure 3)

The ability of the bone marrow derived hMSCs to impact the CFUs of* Staphylococcus aureus* was consistent with the* Pseudomonas aeruginosa* CFU data.* Staphylococcus aureus* at baseline resulted in 27 ± 7 CFUs (Figure 3(a)), whereas treatment of the bacteria with geneticin or hMSC supernatants had 15 ± 5 CFUs or 18 ± 2 CFUs, respectively (*P* < 0.05, *n* = 4 hMSC donor supernatants). When the hMSC supernatants were combined with the geneticin, the impact on* Staphylococcus aureus* growth was additive, resulting in 12 ± 2 CFUs, which was statistically less than either hMSCs supernatants treatment or antibiotic alone (*P* ≤ 0.05 for each, *n* = 4 hMSC supernatants).

The impact of the hMSC on* Staphylococcus aureus* growth rate was different than* Pseudomonas aeruginosa* (Figure 3(b)). It took almost 4 hours before a noticeable change in* Staphylococcus aureus* growth was observed, even when comparing each of the conditions of medium alone, geneticin, hMSC supernatant, and when hMSC supernatant and geneticin were combined. The hMSC supernatants or antibiotic impact on the bacterial growth reached significance at 6 hours and was sustained until 8 hours. During this window of hMSC treatment, the number of viable* Staphylococcus aureus* was significantly decreased with the geneticin (*P* < 0.05, *n* = 4) or hMSC supernatant (*P* < 0.05) which when combined together was additive in antimicrobial effectiveness (*P* < 0.05, *n* = 4 comparing to baseline and each of the conditions alone). This data is consistent with the observation that MSCs are antimicrobial against both* Pseudomonas aeruginosa* and* Staphylococcus aureus* in scenarios of wound healing [9, 25, 26].

#### 3.2.3. *Streptococcus pneumoniae* (Figure 4)

Similar to the* Staphylococcus aureus* and the* Pseudomonas aeruginosa* studies, the bone marrow derived hMSC supernatants decreased* Streptococcus pneumoniae* CFUs (*P* ≤ 0.05) as did the geneticin (Figure 4(a), *P* ≤ 0.05). In addition, like the two other pathogens, there was an enhancing effect of the combination of the hMSC supernatant with the antibiotic on decreasing* Streptococcus pneumoniae* growth (*P* ≤ 0.05). When evaluating the impact of hMSC supernatants on* Streptococcus pneumoniae* survival and growth rate, the response of the pathogen was unique when compared to both the* Pseudomonas aeruginosa* and the* Staphylococcus aureus* (using the same hMSC derived supernatants). The impact of the hMSC supernatants on the* Streptococcus pneumoniae* was not apparent until 1 hour and reached significance at 3 hours (Figure 4(b), *P* < 0.05, *n* = 4 different hMSC supernatants). Further, by 5 hours the geneticin appeared to have become ineffective, while the hMSC supernatants sustained efficacy (duration of the antimicrobial effect). Although the geneticin (*P* < 0.05, *n* = 4) and the hMSC supernatant (*P* < 0.05, *n* = 4) had comparable antimicrobial effectiveness against* Streptococcus pneumoniae*, there was an additive effect (hMSC supernatant and geneticin) on* Streptococcus pneumoniae* ATP production starting at 1 hour, which was consistent with Figure 4(a). However the additive effect of hMSC supernatant and geneticin was short-lived, lasting only until 2 hours posttreatment. There was no statistically sustained benefit of combining the hMSC supernatant and antibiotic on growth rate, suggesting that the hMSC antimicrobial impact on* Streptococcus pneumoniae *growth rate was through different mechanisms than the hMSC supernatant effect on the other two pathogens, especially since the studies were done using the same hMSC supernatant donors, as well as antibiotics. The reasons for the differences are a focus of ongoing studies in the laboratory.

### 3.3. Antibiotic Enhancing Effect

**Figure 5:**
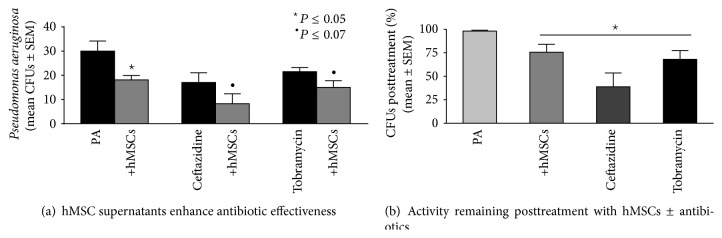
Antibiotic enhancing activity. To determine whether the antibiotic enhancing effects of the bone marrow derived hMSCs were antibiotic specific, we tested the ability of the hMSCs to enhance the impact of other antibiotics which are often used to treat CF: ceftazadine (100 *μ*g/mL) and tobramycin (100 *μ*g/mL). (a) Ceftazadine and tobramycin decreased* Pseudomonas aeruginosa* CFUs (*P* ≤ 0.05, *n* = 3) which were enhanced by the addition of hMSCs derived supernatants (*P* = 0.06 for ceftazadine and *P* = 0.07 for tobramycin). (b) When each experiment is used as its own control for* Pseudomonas aeruginosa* growth (100%), the MSCs and the antibiotics had statistically significant effect on* Pseudomonas* growth (100 *μ*g/mL, *P* ≤ 0.05).

One of the exciting aspects of our studies is the observation that hMSCs may have the potential to serve as an adjunct therapeutic to conventional antibiotics. In addition to geneticin as a broad spectrum antibiotic, we investigated the ability of hMSCs supernatants to enhance the ability of ceftazadine and tobramycin on* Pseudomonas aeruginosa* growth (Figure 5, 100 *μ*g/mL). Ceftazadine and tobramycin are two antibiotics that are commonly used to treat* Pseudomonas aeruginosa* infections in patients with CF. Both ceftazadine and tobramycin were effective at decreasing* Pseudomonas aeruginosa* CFUs (Figure 5(a), *P* ≤ 0.05), as are the hMSC supernatants (*P* ≤ 0.07, *n* = 4 different hMSC donors). Combining respective antibiotic with hMSCs demonstrated an additive effect in decreasing* Pseudomonas aeruginosa* infection which was observed across a variety of antibiotic dosages (data not shown). Evaluating the different experiments for the overall impact on the relative % change comparing antibiotic (either ceftazadine or tobramycin) with hMSC supernatant alone was statistically significant (Figure 5(b), *P* ≤ 0.05, *n* = 4, 100 *μ*g/mL).

### 3.4. Human Bone Marrow Derived MSCs versus Adipose Tissue Derived hMSCs

**Figure 6:**
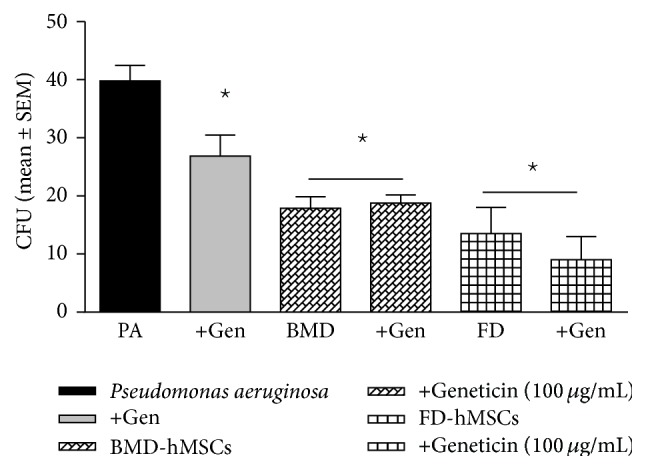
hMSC origin and impact on* Pseudomonas aeruginosa* growth. The availability of different hMSC sources provides the opportunity to explore whether the antimicrobial effectiveness of the hMSC supernatant was dependent on the tissue origin of the hMSC. Like the bone marrow derived hMSCs, hMSCs derived from adipose tissue significantly decreased* Pseudomonas aeruginosa* CFUs (*P* ≤ 0.05) and enhanced geneticin (100 *μ*g/mL) effectiveness (*P* = 0.08, *n* = 3). PA =* Pseudomonas aeruginosa* growth without treatment. Geneticin = treatment with the antibiotic geneticin, +hMSCs = treatment with hMSC derived supernatant, and Both = treatment with both hMSC supernatants and geneticin. hMSC supernatants enhance antibiotic effectiveness against* Pseudomonas aeruginosa*.

To determine if the antimicrobial effectiveness of the hMSCs is dependent on the tissue origin of the hMSCs, we investigated whether hMSCs derived from adipose tissue also demonstrated the potential for antibiotic enhancing activity. As with the bone marrow derived hMSCs, supernatants from adipose tissue derived hMSCs were also antimicrobial (Figure 6, *P* < 0.05, *n* = 3 different donors) decreasing* Pseudomonas aeruginosa* CFUs to levels comparable with the antibiotic control (geneticin). Further, when combining the geneticin with the adipose tissue derived hMSCs, the impact was consistently more potent against* Pseudomonas aeruginosa* than when using bone marrow derived hMSCs in combination with geneticin (*P* ≤ 0.05), which may be related to the donor or to the way in which each type of MSC was isolated and grown. Further, it should be noted that, in these subsets of experiments, the effectiveness of the MSCs augmented the antibiotic sensitivity; however, there was no demonstrated difference between the MSCs supernatant and the combined MSCs + geneticin. In addition, although the FD derived MSCs were antimicrobial and enhanced antibiotic effectiveness compared to geneticin alone, the significant variability minimized the difference of the combined therapeutics when compared to MSC supernatant alone. These observations emphasize the variability with different MSC preparations and the need for optimization.

### 3.5. I-172 versus Baseline Supernatants

**Figure 7:**
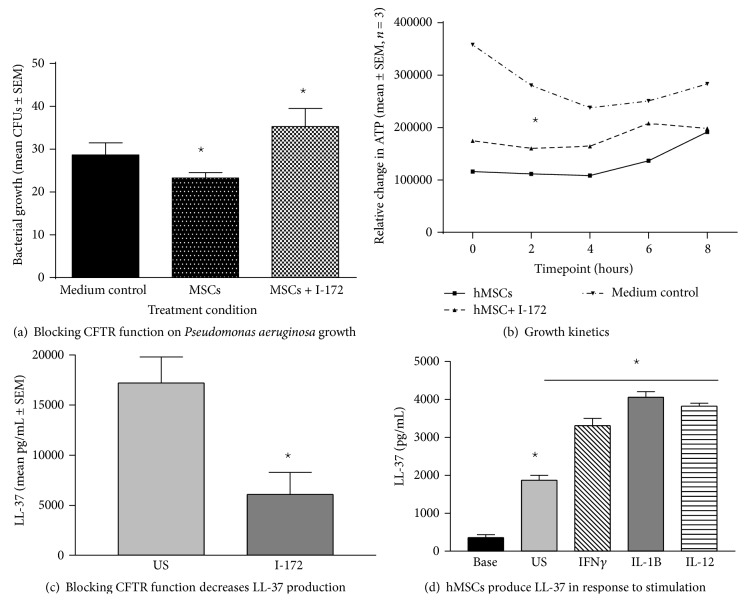
Impact of blocking CFTR function on antimicrobial activity of MSCs. To mimic CF cells, healthy bone marrow derived hMSCs were cultured in the presence and absence of CFTR blocker I-172 (10 *μ*g/mL) without antibiotics for 24 hours. The hMSC supernatants were evaluated for the ability to impact* Pseudomonas aeruginosa* PA CFUs (a) and growth rate (b). Supernatants generated from CFTR deficient hMSCs were more inefficient at decreasing* Pseudomonas aeruginosa* CFUs ((b), *P* ≤ 0.05) and growth rate ((c), *P* ≤ 0.05) than hMSCs without CFTR activity blocked. Further, hMSCs with deficient CFTR activity had less ability to secrete LL-37 ((c), *P* ≤ 0.05) relative to controls. LL-37 production by bone marrow derived hMSCs is decreased when CFTR is blocked but can be increased by treating the cells with a variety of cytokine stimulators. hMSCs stimulated with cytokines IFN*γ* (100 ng/mL), IL-1B (50 ng/mL), and IL-12 (100 ng/mL) secreted significantly more LL-37 than unstimulated controls ((d), *P* ≤ 0.05, *n* = 4 different hMSC preparations).

Our previous data has suggested that bone marrow derived hMSCs deficient in CFTR function produce less LL-37 [8]. To determine whether hMSCs deficient in* Cftr* activity would have decreased antimicrobial activity, we tested supernatants from hMSCs that had been pretreated with the CFTR inhibitor I-172 (Sigma Chemical Co., 10 *μ*g/mL; 48 hours). We confirmed that CFTR deficient hMSCs have less antimicrobial activity than normal hMSCs on* Pseudomonas aeruginosa* CFUs (Figure 7(a), *n* = 4, *P* ≤ 0.05) and alter growth rate (Figure 7(b), *n* = 4, *P* ≤ 0.05). In the* Pseudomonas aeruginosa in vitro *assays, CFUs were evaluated from I-172 treated and untreated hMSC supernatants. hMSC supernatants decreased CFUs from 33 ± 3.5 to 28.5 ± 8.5 (*P* value) when compared to using hMSC supernatants generated in the presence of the CFTR inhibitor (38 ± 16 CFUs). Blocking hMSC CFTR activity also resulted in less efficient impact on decreasing the* Pseudomonas aeruginosa* growth rate. These data confirmed the inefficiency of CFTR deficient hMSC supernatants on decreasing* Pseudomonas aeruginosa* bacterial burden (Figure 7(b), *P* < 0.05, *n* = 4 different hMSC donors) when compared to hMSC supernatants derived from hMSCs cultured without the CFTR inhibitor (*P* < 0.05, *n* = 4 different MSC donors). hMSC supernatants were also evaluated for the presence of the antimicrobial peptide LL-37 since the difference in antimicrobial effectiveness may be related to LL-37 activity. hMSCs which have CFTR blocked with the inhibitor I-172 secreted significantly less LL-37 compared to the hMSCs not treated with the CFTR inhibitor (Figure 7(c); *P* ≤ 0.05, *n* = 5). This data implicates a relationship between LL-37 secretion, CFTR activity, antimicrobial effectiveness, and responsiveness of the hMSCs to bacterial pathogens.

### 3.6. Inducing LL-37

To determine if we could optimize hMSCs for antimicrobial potency, we evaluated the hMSCs for secretion and expression of the antimicrobial defensin LL-37, as well as human beta defensins (hBD) hBD-3 and hBD-2 in response to a variety of effectors. None of the cultures, regardless of whether or not they were stimulated, contained (hBD-2 or hBD-3, (data not shown)) consistent with previous reports [9]. LL-37 is constitutively secreted by bone marrow derived hMSCs. The levels of LL-37 can be enhanced when the hMSCs are stimulated with effectors IFN*γ*, IL-1*β*, or IL-12 (Figure 7(d), *P* ≤ 0.05 for each). Further, the source how the MSCs are grown for clinical use utilizing either serum-free conditions or platelet lysate conditions also had an impact on the* in vivo* antimicrobial efficacy of the hMSCs and production of LL-37. Unlike bone marrow derived hMSCs grown in fetal bovine serum, hMSCs grown under serum-free conditions or platelet lysate conditions produced no detectable LL-37 unless stimulated by LPS (≥1000 pg/mL, *P* ≤ 0.05) which was associated with the overall antimicrobial effectiveness. This may be why certain preparations of the MSCs have better antimicrobial and antibiotic enhancing activity than other preparations as observed in Figure 6; implicate the need for MSC optimization for clinical impact.

To begin to explore the mechanisms by which CFTR regulates LL-37 production, we took advantage of an immortal-mouse derived MSC clone BMC9. These cells are abundant and have an MSC phenotype when grown at 37°C. Further, we could actively quantify mouse* Cftr* using a highly reproducible mouse* Cftr* gene expression assay in our CF Animal CORE Center. The BMC9 cells have 36 ± 14%* Cftr* expression (Ct value of 31 ± 1) compared to intestinal epithelium (Ct value equals 20 ± 3), with our sensitivity and specificity to detect* Cftr* at 40 ± 2 Ct. BMC9 cells cultured with I-172 (10 *μ*g/mL, for 48 hours) expressed 37 ± 13% less mouse mCRAMP (mouse cathelicidin-LL-37 related antimicrobial peptide, mean ± SEM, *n* = 3) than BMC9 cells not cultured with I-172 (*P* ≤ 0.05) [27]. These data suggest that hMSCs express CFTR and that CFTR function impacts the ability of hMSCs to produce LL-37. Studies are ongoing to establish how this might affect clinical impact.

## 4. Discussion

CF patients have increased susceptibility to pulmonary infections with* Pseudomonas aeruginosa* as well as pathogens such as* Staphylococcus aureus *and* Streptococcus pneumoniae* due to the unique environment created by deficient CFTR. The inability to resolve these infections and the ensuing severe inflammatory response is the major cause of morbidity and mortality in CF. Our previous data has shown that hMSCs decrease inflammation and infection in the* in vivo* murine model of CF chronic* Pseudomonas aeruginosa* infection and inflammation. In this paper we demonstrate that hMSCs have a beneficial impact on the* Staphylococcus aureus in vivo* murine model of CF lung infection and inflammation. In addition, we demonstrate that, in both* in vivo* and* in vitro* systems, hMSCs are beneficial in treating infections associated with both gram negative and gram positive pathogens and that the antimicrobial impact of the hMSCs can be associated with the antimicrobial peptide LL-37. The studies presented in this paper also focus on understanding the diversity in the hMSCs antimicrobial effectiveness and whether the hMSC effect is related to changing the overall growth rate of the bacteria depending on the type of pathogen. Further, we demonstrate the ability to optimize the hMSCs and their secreted products based upon source of tissue origin, since stimulation with a variety of stimulators or blocking CFTR activity alters the phenotype of the hMSCs.

Our studies demonstrate that (1) soluble products generated by hMSCs significantly decrease* Pseudomonas aeruginosa*,* Staphylococcus aureus,* and* Streptococcus pneumoniae* CFUs as well as having an impact on the growth rate of these pathogens; (2) the antimicrobial effectiveness of the hMSCs supernatants can enhance the effectiveness of antibiotics used to treat these types of infections; (3) hMSCs produce antimicrobial peptides such as LL-37 which are impacted by the function of CFTR or specific growth medium, which ultimately impacts the hMSC antimicrobial potency (measuring the strength of the hMSC effect) and efficacy (measuring the duration of the hMSC effect); (4) hMSCs derived from bone marrow or adipose tissue are both antimicrobial, suggesting that perhaps all hMSCs, regardless of their tissue origin, can be antimicrobial and could feasibly be optimized for therapeutic applications in infection.

The studies in this paper suggest that MSCs not only impact inflammation and infection but also are diverse at being able to slow the growth rate of a variety of bacteria.* In vivo* the hMSCs have a beneficial preclinical effect in both* Pseudomonas aeruginosa* and* Staphylococcus aureus *infection and inflammation murine models of CF. These studies imply that the hMSCs have the capacity to manage both gram negative and gram positive infections in CF, despite different levels of potency and efficacy. Further, the antimicrobial effectiveness is dependent on how the hMSCs are cultured which are likely different for each of the pathogens. Additionally, the antimicrobial and antibiotic enhancing activity of the MSCs appears to be dependent on how the MSCs are grown, donor variability, as well as the pathogen.

In terms of mechanisms related to the antimicrobial function of the hMSCs there are at least two ways in which the hMSC supernatants are impacting bacterial growth and activity. The studies in this paper suggest that the hMSCs are directly slowing down the growth activity of the bacteria resulting in a lower bacteria burden than what would otherwise result without the presence of the hMSC supernatants. The slowed growth and fewer bacteria can induce a window of opportunity for the antibiotics as well as the host's immune system to help resolve the infection. The concept of the hMSC supernatant impact on the bacteria can be broken down into at least two possible mechanisms. The first is by decreasing the overall bacterial burden or ability of the bacteria to overcome the antibiotic concentration. This allows for more efficient antibiotic effectiveness because the overall number of bacteria is more easily managed by the antibiotic treatment. The rate of the bacterial growth and the potency of the hMSC supernatant would be variables of the antimicrobial effect, which probably explains the differences in the overall kinetics of the hMSC supernatant impact on* Pseudomonas aeruginosa*,* Staphylococcus aureus,* and* Streptococcus pneumoniae*. This function of the hMSCs may be especially important in scenarios of sepsis, as well as CF, in which the bacteria burden, bacteria death by antibiotics, and release of proinflammatory stimulators contribute to the tissue damage [28, 29]. In sepsis studies, the battle between prevention of bacterial overgrowth, the intensity of how the bacteria are killed, and the efficiency of the process is hypothesized to define the progression to death [29, 30].

The second piece of the important antimicrobial effectiveness of the hMSCs is the ability of the stem cells to secrete antimicrobial peptides including the peptide LL-37 [8, 9]. Like other antimicrobial peptides, LL-37 mediates its effects by softening the bacterial cell wall and allowing increased sensitivity to host and antibacterial agents [31]. Since the impact of the LL-37 is on the bacterial wall, the potency of the hMSC antimicrobial effectiveness may be related to the type of pathogen [32, 33] which explains why the hMSC supernatants had different effects on* Staphylococcus aureus* and* Streptococcus pneumoniae*. For these two different pathogens it took longer to have an antimicrobial impact than when treating the* Pseudomonas aeruginosa*. This may be relative to the proportions and types of gram negative and gram positive organisms and the efficiency of both MSCs and LL-37 as well as a variety of other potential antimicrobial peptide products [34, 35]. Further, the impact of the hMSC supernatants on improving geneticin sensitivity was not as potent, which could be related to the delayed impact of the hMSCs on the* Staphylococcus aureus* and* Streptococcus pneumoniae* growth kinetics. The action of the hMSCs and production of LL-37 may also be additive with the host response. hMSCs have the capacity to change the localize milieu eliciting a host response appropriate for the scenario at hand. Further, antimicrobial potency including additional sources of LL-37 may be induced in the host in response to the hMSCs. The hMSCs may induce an additive boost of antimicrobial efficiency harnessing both hMSCs and host effectiveness [31, 36, 37].

In this paper, we show that the production of LL-37 is related to the antimicrobial effectiveness of the hMSCs supernatants and that when the LL-37 is downregulated (when CFTR is blocked by the inhibitor I-172), the antimicrobial effectiveness of the supernatants becomes less efficient. There have been previous reports suggesting that LL-37 in scenarios of* Pseudomonas aeruginosa* infection or methicillin resistant* Staphylococcus aureus* and group B-*Streptococcus pneumoniae* (*Streptococcus pyrogenes*) may contribute to antibiotic sensitivity and regulate the production of quorum sensing molecules [32, 33] associated with the antimicrobial effectiveness of LL-37. Our studies were done with a clinical strain of mucoid* Pseudomonas aeruginosa*, non-MRSA* Staphylococcus aureus*, and* Streptococcus pneumoniae* with and without antibiotics which implies that close attention must be made in understanding hMSC and LL-37 therapy in scenarios of complex infections and source of pathogens. Further, additional properties of the hMSC supernatants may also be associated with antimicrobial potency and efficacy, including other antimicrobial molecules, although we did not detect the presence of defensins hBD-3 or hBD-2. This is the focus of on-going work in our laboratory. Our studies also suggest that recombinant LL-37 along with antibiotics may be a reasonable therapeutic for CF; however the high chloride environment of the CF lung does not sustain LL-37 functional activity [35, 38]. Repeated dosages of recombinant LL-37 would have to be nebulized to keep active LL-37 in the lung. Based upon our studies, however, hMSCs may provide a unique therapeutic alternative as a continuous source of LL-37, to contribute to their environmental milieu [33, 34].

Another important observation from these studies is the similarities between the bone marrow derived hMSC supernatants and the adipose tissue derived hMSC supernatants in antimicrobial activity. The tissue origin of the hMSCs is likely to reflect where the MSCs reside, their initial niche of differentiation and surrounding microenvironment [39, 40]. Our data shows that although bone marrow derived and adipose derived hMSCs come from different environmental niches, both sources of hMSCs possess important antimicrobial properties. These studies support that hMSCs, whether they are derived from bone marrow or adipose tissue, have the potential to have imminent therapeutic impact in scenarios of infection and inflammation.

Our data also suggests that, in terms of hMSC therapy in CF, the patient's own cells may not be as efficient at antimicrobial activity as MSC derived from healthy individuals. hMSCs with deficient CFTR function produce less LL-37, which has the capacity to contribute to inefficient* Pseudomonas aeruginosa* killing. This suggests that the patients' own cells may not be as efficient in the host response to infection. Further, whether deficient hMSC LL-37 is part of the pathophysiology related to the inability of the CF patients to resolve infection and whether supplemental therapeutics with hMSCs from healthy donors may be beneficial remain to be determined. Our laboratories are actively studying this observation since it has implications not only for CF but also for other diseases. These data also suggest that environment and phenotype are important factors in determining the overall beneficial effect of hMSCs in resolving bacterial infections. The optimal conditions for generating an hMSC phenotype with the most efficient antimicrobial and antibiotic enhancing effectiveness have yet to be identified and are the focus of high-throughput studies to identify the hMSC response versus function for clinical applications.

From these studies, we can conclude that (1) hMSC therapy improves outcomes in CF lung infection with both* Pseudomonas aeruginosa* and* Staphylococcus aureus in vivo* determined by the murine model of CF lung infection and inflammation; (2) hMSC supernatants significantly decreased the growth of* Pseudomonas aeruginosa, Staphylococcus aureus, *and* Streptococcal pneumoniae in vitro*; (3) both sources of MSCs have the capacity to have antimicrobial and antibiotic enhancing activity which is dependent on the donor as well as growth conditions in the preparations of the hMSC; (4) hMSCs secreted bioactive molecules, including LL-37, but not hBD-3 or hBD-2, which is consistent with antimicrobial effectiveness; (5) the impact of hMSCs on antibiotics appears to be concentration and time course dependent, as well as MSC source dependent and pathogen specific; (6) growth conditions significantly impact the overall antimicrobial impact such as altering MSC-CFTR activity or stimulating with a variety of cytokines when compared to MSCs not treated with any stimulant; (7) hMSCs with deficient CFTR activity are not as efficient at handling infection as hMSCs with sufficient CFTR activity when evaluated using* in vitro* antimicrobial assays monitoring pathogen growth kinetics and CFUs.

## 5. Summary

The studies outlined in this paper demonstrate the diverse antimicrobial and antibiotic enhancing potency of hMSCs and their products. MSCs as therapeutic powerhouses may require optimization for the greatest clinical impact. Further, the studies in this paper emphasize the significant clinical potential of MSCs for treating infections like those associated with cystic fibrosis chronic lung disease and support our Phase I Clinical Trial investigating the safety of hMSCs in patients with CF.

## References

[1] Chmiel J. F., Konstan M. W. (2005). Anti-inflammatory medications for cystic fibrosis lung disease: selecting the most appropriate agent. *Treatments in Respiratory Medicine*.

[2] Nichols D. P., Konstan M. W., Chmiel J. F. (2008). Anti-inflammatory therapies for cystic fibrosis-related lung disease. *Clinical Reviews in Allergy and Immunology*.

[3] Chmiel J. F., Konstan M. W. (2007). Inflammation and anti-inflammatory therapies for cystic fibrosis. *Clinics in Chest Medicine*.

[4] Weiss D. J., Berberich M. A., Borok Z. (2006). Adult stem cells, lung biology, and lung disease. NHLBI/Cystic Fibrosis Foundation Workshop. *Proceedings of the American Thoracic Society*.

[5] Caplan A. I. (2009). Why are MSCs therapeutic? New data: new insight. *Journal of Pathology*.

[6] Abdallah B. M., Kassem M. (2008). Human mesenchymal stem cells: from basic biology to clinical applications. *Gene Therapy*.

[7] Caplan A. I., Dennis J. E. (2006). Mesenchymal stem cells as trophic mediators. *Journal of Cellular Biochemistry*.

[8] Bonfield T. L., Lennon D., Ghosh S. K., DiMarino A. M., Weinberg A., Caplan A. I. (2013). Cell based therapy aides in infection and inflammation resolution in the murine model of cystic fibrosis lung disease. *Stem Cell Discovery*.

[9] Krasnodembskaya A., Song Y., Fang X. (2010). Antibacterial effect of human mesenchymal stem cells is mediated in part from secretion of the antimicrobial peptide LL-37. *Stem Cells*.

[10] Bragonzi A., Farulla I., Paroni M. (2012). Modelling co-infection of the cystic fibrosis lung by Pseudomonas aeruginosa and Burkholderia cenocepacia reveals influences on biofilm formation and host response. *PLoS ONE*.

[11] Sagel S. D., Chmiel J. F., Konstan M. W. (2007). Sputum biomarkers of inflammation in cystic fibrosis lung disease. *Proceedings of the American Thoracic Society*.

[12] Bonfield T. L., Nolan M. T., Lennon D. P., Caplan A. I. (2010). Defining human mesenchymal stem cell efficacy in vivo. *Journal of Inflammation*.

[13] Lennon D. P., Caplan A. I. (2006). Isolation of human marrow-derived mesenchymal stem cells. *Experimental Hematology*.

[14] Chen Y.-T., Sun C.-K., Lin Y.-C. (2011). Adipose-derived mesenchymal stem cell protects kidneys against ischemia-reperfusion injury through suppressing oxidative stress and inflammatory reaction. *Journal of Translational Medicine*.

[15] LeVine A. M., Kurak K. E., Wright J. R. (1999). Surfactant protein-a binds group B streptococcus enhancing phagocytosis and clearance from lungs of surfactant protein-a-deficient mice. *American Journal of Respiratory Cell and Molecular Biology*.

[16] Heeckeren A., Walenga R., Konstan M. W., Bonfield T., Davis P. B., Ferkol T. (1997). Excessive inflammatory response of cystic fibrosis mice to bronchopulmonary infection with *Pseudomonas aeruginosa*. *Journal of Clinical Investigation*.

[17] Cash H. A., Woods D. E., McCullough B., Johanson W. G., Bass J. A. (1979). A rat model of chronic respiratory infection with *Pseudomonas aeruginosa*. *American Review of Respiratory Disease*.

[18] Archer N. K., Mazaitis M. J., Costerton J. W., Leid J. G., Powers M. E., Shirtliff M. E. (2011). *Staphylococcus aureus* biofilms: properties, regulation, and roles in human disease. *Virulence*.

[19] Ghosh S. K., Gerken T. A., Schneider K. M., Feng Z., McCormick T. S., Weinberg A. (2007). Quantification of human *β*-defensin-2 and -3 in body fluids: application for studies of innate immunity. *Clinical Chemistry*.

[20] Kawsar H. I., Ghosh S. K., Hirsch S. A., Koon H. B., Weinberg A., Jin G. (2010). Expression of human beta-defensin-2 in intratumoral vascular endothelium and in endothelial cells induced by transforming growth factor beta. *Peptides*.

[21] Simon R., Radmacher M. D., Dobbin K. (2002). Design of studies using DNA microarrays. *Genetic Epidemiology*.

[22] Simon B. A., Easley R. B., Grigoryev D. N. (2006). Microarray analysis of regional cellular responses to local mechanical stress in acute lung injury. *American Journal of Physiology: Lung Cellular and Molecular Physiology*.

[23] Drumm M. L., Konstan M. W., Schluchter M. D. (2005). Genetic modifiers of lung disease in cystic fibrosis. *The New England Journal of Medicine*.

[24] Schluchter M. D., Konstan M. W., Drumm M. L., Yankaskas J. R., Knowles M. R. (2006). Classifying severity of cystic fibrosis lung disease using longitudinal pulmonary function data. *American Journal of Respiratory and Critical Care Medicine*.

[25] Guerra A. D., Cantu D. A., Vecchi J. T., Rose W. E., Hematti P., Kao W. J. (2015). Mesenchymal stromal/stem cell and minocycline-loaded hydrogels inhibit the growth of *Staphylococcus aureus* that evades immunomodulation of blood-derived leukocytes. *The AAPS Journal*.

[26] Matthay M. A., Goolaerts A., Howard J. P., Lee J. W. (2010). Mesenchymal stem cells for acute lung injury: preclinical evidence. *Critical Care Medicine*.

[27] Kovach M. A., Ballinger M. N., Newstead M. W. (2012). Cathelicidin-related antimicrobial peptide is required for effective lung mucosal immunity in gram-negative bacterial pneumonia. *Journal of Immunology*.

[28] Lever A., Mackenzie I. (2007). Sepsis: definition, epidemiology, and diagnosis. *British Medical Journal*.

[29] Napolitano L. M. (2005). Immune stimulation in sepsis: to be or not to be?. *Chest*.

[30] Lyn-Kew K., Standiford T. J. (2008). Immunosuppression in sepsis. *Current Pharmaceutical Design*.

[31] Golec M. (2007). Cathelicidin LL-37: LPS-neutralizing, pleiotropic peptide. *Annals of Agricultural and Environmental Medicine*.

[32] Sakoulas G., Guram K., Reyes K., Nizet V., Zervos M. (2014). Human cathelicidin LL-37 resistance and increased daptomycin MIC in methicillin-resistant *Staphylococcus aureus* strain USA600 (ST45) are associated with increased mortality in a hospital setting. *Journal of Clinical Microbiology*.

[33] Strempe N., Neidig A., Nusser M. (2013). Human host defense peptide LL-37 stimulates virulence factor production and adaptive resistance in *Pseudomonas aeruginosa*. *PLoS ONE*.

[34] Scott A., Weldon S., Buchanan P. J. (2011). Evaluation of the ability of LL-37 to neutralise LPS in vitro and ex vivo. *PLoS ONE*.

[35] Brown K. L., Poon G. F. T., Birkenhead D. (2011). Host defense peptide LL-37 selectively reduces proinflammatory macrophage responses. *Journal of Immunology*.

[36] Méndez-Samperio P. (2010). The human cathelicidin hCAP18/LL-37: a multifunctional peptide involved in mycobacterial infections. *Peptides*.

[37] Auletta J. J., Eid S. K., Wuttisarnwattana P. (2014). Human mesenchymal stromal cells attenuate graft-versus-host disease and maintain graft-versus-leukemia activity following experimental allogeneic bone marrow transplantation. *STEM CELLS*.

[38] Chen C. I.-U., Schaller-Bals S., Paul K. P., Wahn U., Bals R. (2004). *β*-defensins and LL-37 in bronchoalveolar lavage fluid of patients with cystic fibrosis. *Journal of Cystic Fibrosis*.

[39] Zagoura D. S., Trohatou O., Bitsika V. (2013). AF-MSCs fate can be regulated by culture conditions. *Cell Death and Disease*.

[40] Soleymaninejadian E., Pramanik K., Samadian E. (2012). Immunomodulatory properties of mesenchymal stem cells: cytokines and factors. *American Journal of Reproductive Immunology*.

